# The Effect of Ionizing Irradiation on the Autotaxin-Lysophasphatidic Acid Axis and Interleukin-6/8 Secretion in Different Breast Cancer Cell Lines

**DOI:** 10.3390/jpm14090968

**Published:** 2024-09-12

**Authors:** Theresa Promny, Isabell Scherrer, Sheetal Kadam, Rafael Schmid, Tina Jost, Luitpold V. Distel, Andreas Arkudas, Raymund E. Horch, Annika Kengelbach-Weigand

**Affiliations:** 1 Department of Plastic and Hand Surgery and Laboratory for Tissue Engineering and Regenerative Medicine, University Hospital Erlangen, Friedrich-Alexander University Erlangen-Nürnberg (FAU), 91054 Erlangen, Germany; isabell.scherrer@uk-erlangen.de (I.S.); sheetal.kadam@uk-erlangen.de (S.K.); rafael.schmid@uk-erlangen.de (R.S.); andreas.arkudas@uk-erlangen.de (A.A.); raymund.horch@uk-erlangen.de (R.E.H.); annika.kengelbach-weigand@uk-erlangen.de (A.K.-W.); 2 Department of Radiation Oncology, University Hospital Erlangen, Friedrich-Alexander-Universität Erlangen-Nürnberg, 91054 Erlangen, Germany; tina.jost@uk-erlangen.de (T.J.); luitpold.distel@uk-erlangen.de (L.V.D.)

**Keywords:** lysophosphatidate signaling, inflammatory mediators, LPA receptors, ATX, adipose-derived stem cells, ADSC

## Abstract

Background: The Autotaxin (ATX)-lysophosphatidic acid (LPA) axis is involved in decreasing radiation sensitivity of breast tumor cells. This study aims to further elucidate the effect of irradiation on the ATX-LPA axis and cytokine secretion in different breast cancer cell lines to identify suitable breast cancer subtypes for targeted therapies. Methods: Different breast cancer cell lines (MCF-7 (luminal A), BT-474 (luminal B), SKBR-3 (HER2-positive), MDA-MB-231 and MDA-MB-468 (triple-negative)) and the breast epithelial cell line MCF-10A were irradiated. The influence of irradiation on LPA receptor (LPAR) expression, ATX expression, and Interleukin (IL)-6 and IL-8 secretion was analyzed. Further, the effect of IL-6 and IL-8 on ATX expression of adipose-derived stem cells (ADSC) was investigated. Results: Irradiation increased ATX and LPAR2 expression in MDA-MB-231 cells. Additionally, IL-6 secretion was enhanced in MDA-MB-231, and IL-8 secretion in MDA-MB-231 and MDA-MB-468. Stimulation of ADSC with IL-6 and IL-8 increased ATX expression in ADSC. Conclusions: Targeting ATX or its downstream signaling pathways might enhance the sensitivity of triple-negative breast cancer cells to radiation. Further exploration of the interplay between irradiation, the ATX-LPA axis, and inflammatory cytokines may elucidate novel pathways for overcoming radioresistance and improving individual treatment outcomes.

## 1. Introduction

Radiotherapy represents an integral component in the multimodal treatment of breast cancer. In multiple cases, adjuvant radiation is applied after a mastectomy or breast-conserving therapy to eliminate residual breast cancer cells and successfully reduces the risk of local and regional recurrences [1]. However, some patients still develop locoregional recurrence following radiotherapy. This may be due to residual disease, aggressive tumor biology, or intrinsic or acquired resistance of breast cancer cells to radiotherapy. Therefore, investigations on improving outcomes from radiotherapy have been conducted over decades. While the initial focus was on the molecular mechanisms of the cancer cell itself, research within the last years has tended to focus on interactions between the tumor and its surrounding tissue, the tumor microenvironment.

One of the signaling pathways identified to play a role in radiotherapy resistance is the Autotaxin (ATX)-lysophosphatidate (LPA) axis [2,3,4,5,6]. ATX is a plasma lysophospholipase D that hydrolyzes lysophosphatidylcholine (LPC) into the bioactive phospholipid LPA [7,8]. Aside from its physiological properties, LPA is also attributed an important role in the progression and metastasis of different types of cancer, including breast cancer [9,10,11,12,13,14]. LPA acts through at least six specific G-protein-coupled LPA receptors (LPAR1–6) on the cell membrane [15,16]. Breast cancer is known to be a very heterogeneous tumor entity, and the expression of the different LPAR, and thus, the influence of the ATX-LPA axis, varies depending on the breast cancer subtype or cell line [17,18,19]. Although ATX levels are increased in breast tumors, previous studies revealed that cancer cells are only minor producers of ATX compared to the adjacent tissue [20,21,22]. Adipose-derived stem cells (ADSC) from healthy tissue and particularly, ADSC adjacent to tumor cells, express high levels of ATX [20,21]. Benesch et al. suggested a vicious cycle in which tumor-induced inflammation in mammary adipose tissue stimulates ATX secretion and cancer progression [22]. Further, there is evidence that increased LPA signaling induces resistance to chemotherapy and radiotherapy [2]. Previous studies showed that LPA activates signaling pathways promoting cell survival and DNA damage repair [23,24]. This might support cancer cells to recover from radiation-induced damage. Especially, the activation of LPAR2 was linked to the antiapoptotic effect of LPA by diminishing the mitochondrial apoptosis cascade [2,24]. Meng et al. suggested a radiation-induced wound-healing response in adipose tissue that involves ATX-LPA signaling by increasing the levels of ATX, LPAR (LPAR1 and LPAR2) and other inflammatory mediators [25,26]. This LPA-mediated chronic activation of inflammatory pathways in the tumor microenvironment could promote the radioresistance of breast cancer cells and thus limit the efficacy of the treatment.

Understanding the mechanisms underlying radiotherapy resistance in different breast cancer types is essential for the development of new personalized therapeutic strategies to improve the survival rate and personalized treatment plans. Hence, this study aims to further elucidate the effect of irradiation on the ATX-LPA axis and cytokine secretion in different breast cancer cell lines. Further, the influence of interleukins on ATX expression in ADSC was investigated.

## 2. Materials and Methods

### 2.1. The Cell Lines and Cell Culture

MCF-7 (American Type Culture Collection, ATCC (Manassas, VA, USA), luminal A) and MDA-MB-231 (ATCC HTB-26, triple-negative, mesenchymal-like) were cultured in DMEM (Gibco, Life Technologies, Carlsbad, CA, USA), 10% fetal bovine serum (FBS; Biochrom AG, Berlin, Germany), 1% non-essential amino acids (Gibco, Life Technologies, Carlsbad, CA, USA), and 1% L-glutamine (Sigma-Aldrich, St. Louis, MO, USA). SKBR3 (ATCC HTB-30, HER2-positive) were cultivated in McCoy’s 5a Modified Medium (Thermo Fisher Scientific Inc., Waltham, MA, USA) with the addition of 10% FBS. MDA-MB-468 (ATCC HTB-132, triple-negative, basal-like) were cultured in DMEM/F12 (Biochrom AG, Berlin, Germany), 10% FBS, and BT-474 (ATCC HTB-20, luminal B) were cultured in Hybri-Care Medium (ATCC), 10% FBS, and 1.5 g/l NaHCO3 (Sigma Aldrich, St. Louis, MO, USA). The mammary epithelial cell line MCF-10A (ATCC CRL-10317) was cultivated in Mammary Epithelial Cell Growth Medium (PromoCell GmbH, Heidelberg, Germany) enriched with 100 ng/mL cholera toxin (Sigma Aldrich, St. Louis, MO, USA), 5 µg/mL insulin, 0.5 µg/mL hydrocortisone, 10 ng/mL epidermal growth factor (EGF) (PromoCell GmbH, Heidelberg, Germany), and 4 µL/mL bovine pituitary extract (BPE) (PromoCell GmbH, Heidelberg, Germany). The cell culture medium of all breast cancer cell lines contained 1% penicillin/streptomycin (Sigma-Aldrich, St. Louis, MO, USA).

An ASC/TERT1 (human-adipose-tissue-derived telomerase-immortalized mesenchymal stem cell line) was purchased from Evercyte (Evercyte GmbH, Vienna, Austria) and cultivated in Endothelial Cell Growth Medium (EGM)-2 BulletKit (Lonza Group AG, Basel, Switzerland). This culture system contains Endothelial Cell Basal Medium-2 (EBM-2) and EGM-2 SingleQuots Supplements (both from Lonza Group AG, Basel, Switzerland) with the addition of 200 µg/mL Geneticin (Gibco, Life Technologies, Carlsbad, CA, USA) and 2% fetal calf serum superior (Sigma Aldrich, St. Louis, MO, USA).

Cells were cultivated at 37 °C and 5% CO_2_ and the medium was changed every 2–3 days. In the culture medium, 10% FBS, or 4 µL/mL BPE, respectively, was replaced by 0.2% fatty-acidfree bovine serum albumin (BSA; Sigma Aldrich) for all experimental groups.

**Figure 1:**
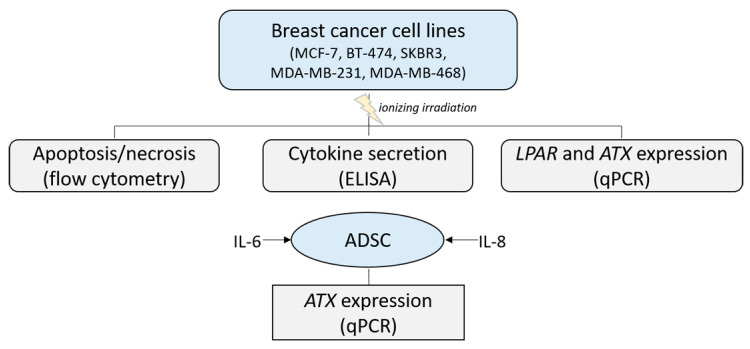
An overview of the performed experiments. The influence of ionizing radiation on the apoptosis/necrosis of different breast cancer cell lines was analyzed using flow cytometry. The lysophosphatidic acid receptor (LPAR) and autotaxin (ATX) expression in the cell lines were detected with a quantitative real-time PCR (qPCR), and cytokine secretion was analyzed using enzyme-linked immunosorbent assay (ELISA) measurements. Subsequently, the influence of enhanced secreted interleukin (IL-)6 and 8 on the expression of ATX in adipose-derived stem cells (ADSC) was investigated.

Figure 1 provides an overview of the performed experiments.

### 2.2. Irradiation

Irradiation was performed with ionizing radiation at a voltage of 120 kV and a 2 mm aluminum filter using an Isovolt Titan 160 X-ray generator (GE Sensing & Inspection Technologies, Ahrensburg, Germany) and a focus-field distance of 21 cm. Irradiation doses of 1 × 2 Gy or 1 × 5 Gy were administered at a dose rate of 2 Gy per minute. The control used was 0 Gy.

### 2.3. Flow Cytometry for the Analysis of Apoptosis and Necrosis

Breast cancer cells, mammary epithelial cells, and supernatant were collected 48 h after irradiation with 2 Gy and 5 Gy (control 0 Gy) and stained with 10 µL of a 1:1 mixture of 7-amino-actinomycin D (7-AAD; BD Biosciences) and Annexin V-APC (BD Biosciences, Heidelberg, Germany) for 30 min on ice and light protected. Cell suspensions were put into 96-well plates to investigate apoptosis and necrosis via the Cytoflex flow cytometer (Cytoflex, Beckman Coulter, Brea, CA, USA). Cells without any staining served as a negative control; cells treated with 56 °C for 20 min were used as a positive control. Data was analyzed with the FlowJo™ Analysis Software v10 (FlowJo LCC, BD Biosciences, Ashland, OR, USA). Cells stained by Annexin V-APC were classified as apoptotic cells. Annexin V-APC-positive and 7AAD-positive cells were defined as necrotic cells. Cells with no staining (Annexin V-APC-negative and 7AAD-negative) were classified as viable cells. The experiments were performed in technical triplicate and in three replicate experiments.

### 2.4. Quantitative Real-Time PCR

The 3 × 10^4^ cells (MDA-MB-468, MDA-MB-231, MCF-7, BT-474, MCF-10A) or 6 × 10^5^ cells (SKBR3), respectively, were seeded in 12-well plates with 1 mL of their standard medium. After incubation for 24 h, the wells were irradiated with 0, 2, and 5 Gy. Right after irradiation, the medium was changed. The mRNA expression of *LPAR1*, *2*, *3*, and *6* and *ATX* was analyzed 48 h after ionizing radiation on the mRNA level. An extraction of RNA was performed with the RNeasy Mini Kit (Qiagen, Hilden, Germany). Reverse transcription into cDNA was followed by using the QuantiTect Reverse Transcription Kit with a DNase I incubation (Qiagen). A quantitative real-time PCR was completed with the SsoAdvanced Universal SYBR Green Supermix (Bio-Rad Laboratories, Hercules, CA, USA) in a Light Cycler (Bio-Rad CFX96). All kits were used following the manufacturers’ instructions. The measured transcript levels were normalized to the housekeeping gene Tyrosine 3-monooxygenase/tryptophan 5-monooxygenase activation protein, zeta (*YWHAZ*) with the 2^−ΔΔCT^ method. The samples were tested in technical triplicate and a PCR was conducted in three independent experiments for 5 Gy, and in technical triplicate and in two replicate experiments for 2 Gy. Primers (Table 1) were designed using the NCBI gene database and purchased from Sigma-Aldrich.

### 2.5. Enzyme-Linked Immunosorbent Assay (ELISA) Measurements

Secretion of IL-6 and IL-8 after irradiation was analyzed with ELISA measurements by using the IMMULITE 1000 Immunoassay System (Siemens, Munich, Germany). The 3 × 10^4^ cells (MDA-MB-468, MDA-MB-231, MCF-7, BT-474, MCF-10A) or 6 × 10^5^ cells (SKBR3), respectively, were seeded in 12-well plates with 1 mL of their standard medium and incubated for 24 h. After irradiation, the medium was changed. Cell culture supernatants were collected from all cell lines 48 h after irradiation. The supernatants were used for the ELISA measurements for the determination of IL-6 and for IL-8 secretions according to the standard instructions. In an exploratory test, 2 and 5 Gy showed similar results, so the focus for the detailed analysis of cytokine secretion was placed on 5 Gy. The experiments were performed in technical triplicate and in at least three replicate experiments (*n* = 3 replicate experiments for MDA-MB-231 IL-6, IL-8, and MDA-MB-468 IL-8; *n* = 5 replicate experiments for MDA-MB-468 IL-6).

### 2.6. The Stimulation of ADSCs

The 2 × 10^5^ ASC/TERT1 were seeded in 6-well plates. After 24 h, when confluency was around 70%, cells were washed with phosphate buffered saline (PBS). After a PBS wash, the ADSC were stimulated with 0 ng/mL, 1 ng/mL, and 50 ng/mL concentration of IL-6 and IL-8 in a standard medium containing 0.2% fatty-acid-free BSA for 24 h. After 24 h, the *ATX* mRNA expression was analyzed with a quantitative real-time PCR, as described above in Section 2.4. The experiments were performed in technical triplicate and in three replicate experiments.

### 2.7. Statistics

A statistical analysis of the flow cytometry and qPCR experiments was performed using the Kruskal–Wallis test, followed by the Dunn’s test for a post-hoc analysis (GraphPad Prism version 9 for Windows; La Jolla, CA, USA). Differences in survival rates between cell lines were also tested using the Kruskal–Wallis test, followed by the Dunn’s test for a post-hoc analysis. A statistical analysis of cytokine secretion was conducted using the Mann–Whitney U test; the asymptotic significance was used (SPSS v.21.0 Software/IBM, Armonk, NY, USA). The error bars in the graphs represent the standard deviation (SD) of the data set. The graphs were designed with GraphPad (GraphPad Prism version 9). A *p*-value ≤ 0.05 was considered significant.

## 3. Results

### 3.1. 5 Gy Irradiation Reduced Cell Survival Rates

**Figure 2:**
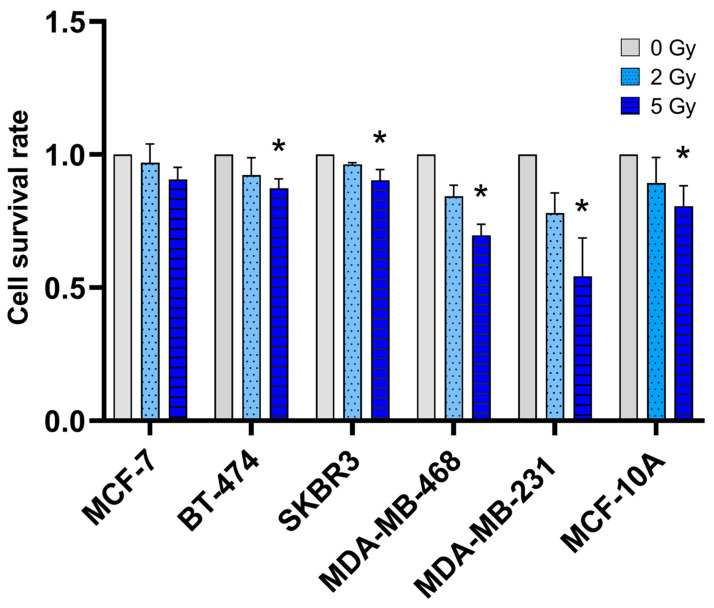
Effect of ionizing radiation on cell survival in different breast cancer cells analyzed with flow cytometry. The columns show the ratio of live cells 48 h after irradiation with 2 Gy and 5 Gy compared to the ratio of live cells of the non-irradiated control group (0 Gy was set as 1). Values are presented as a mean ± SD. * *p* ≤ 0.05.

The effect on cell survival in different breast (cancer) cell lines 48 h after irradiation with 2 Gy and 5 Gy was analyzed with flow cytometry (Figure 2). All cell lines showed significantly reduced cell survival rates after 5 Gy irradiation, except for the MCF-7 cells (BT-474: *p* = 0.03, SKBR3: *p* = 0.01, MDA-MB-468: *p* = 0.01, MDA-MB-231: *p* = 0.01, and MCF-10A: *p* = 0.03). However, the response of different breast cancer cell lines to irradiation varied. While MCF-7, BT-474, and SKBR3 revealed similar cell survival rates to the healthy mammary breast cell line MCF-10A, the triple-negative breast cancer cell lines MDA-MB-468 and MDA-MB-231 showed a non-significant trend to be more responsive to irradiation and showed lower cell survival rates after irradiation.

### 3.2. LPAR Expression of Cell Lines after Irradiation

**Figure 3:**
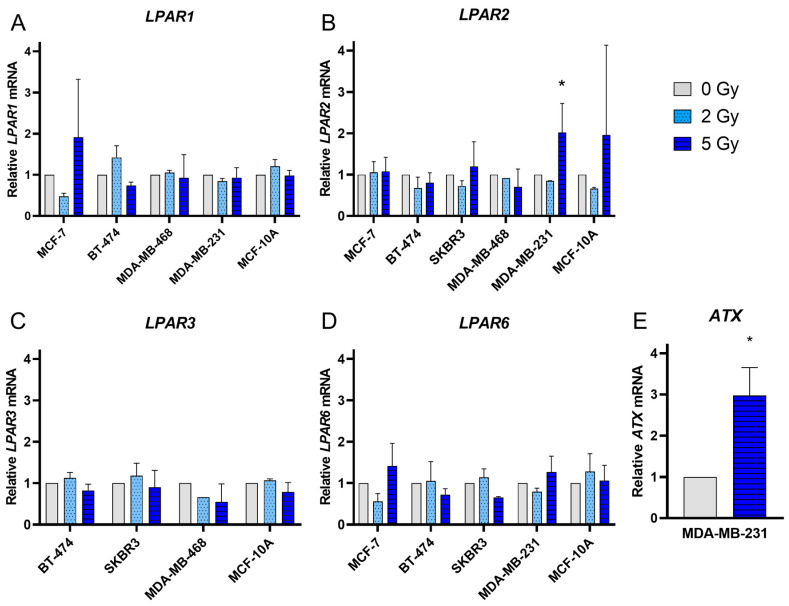
Alterations in the *LPAR1–3* (**A**–**C**), *LPAR6* (**D**) and *ATX* (**E**) mRNA expression of different breast cancer cell lines after irradiation with 2 Gy and 5 Gy compared to receptor expression of non-irradiated cells. Columns show the mean relative mRNA expression (*y*-axis) in different cell lines compared to *YWHAZ* and to the corresponding control group (*x*-axis). *ATX* mRNA expression (**E**) could only be detected for the MDA-MB-231 cells. Values were calculated using the 2^−ΔΔCT^ method, where 2^−ΔΔCT^ of control = 1. * *p* < 0.05.

The effects of irradiation on the mRNA expression of *LPAR1–3* and *LPAR 6* were analyzed at 48 h after irradiation for the different breast cancer cell lines. Exposure to 2 Gy and 5 Gy irradiation did not lead to a significant alteration of mRNA expression in most of the cell lines and for most of the *LPAR* (Figure 3A–D). Enhanced levels of *LPAR* could only be observed for *LPAR2* in the triple-negative cell line MDA-MB-231 after 5 Gy irradiation (*p* = 0.004). It is known that the baseline expression for *LPAR* varies considerably depending on the breast cancer cell line, and some cell lines do not express certain receptors at all [17,21]. In the present experiment, a *LPAR1* expression was not detectable for the SKBR3 cells, and a *LPAR3* expression could not be measured in the MDA-MB-231 cells, and only in one out of three experiments in the MCF-7 cells. A *LPAR6* expression was not detected in the MDA-MB-468 cells.

### 3.3. ATX Expression Was Significantly Increased in the MDA-MB-231 Cells after 5 Gy Irradiation

The ATX-LPA signaling pathway plays an important role in various breast cancer cell lines. However, *ATX* expression is low in breast cancer cell lines and is instead secreted mainly by the tumor microenvironment. Hence, to study the effect of irradiation on *ATX* expression in different breast cancer cell lines, *ATX* mRNA expression was analyzed 48 h after 2 and 5 Gy irradiation. In MDA-MB-231, there was a significant 2.9-fold increase in *ATX* mRNA expression after 5 Gy irradiation compared to 0 Gy (*p* = 0.04; Figure 3E), while 2 and 5 Gy irradiation in other cell lines did not show any significant increase in *ATX* mRNA levels.

### 3.4. Irradiation Leads to Higher Levels of IL-6 Secretion in MDA-MB-231 and Higher Levels of IL-8 Secretion in MDA-MB-468 and MDA-MB-231

**Figure 4:**
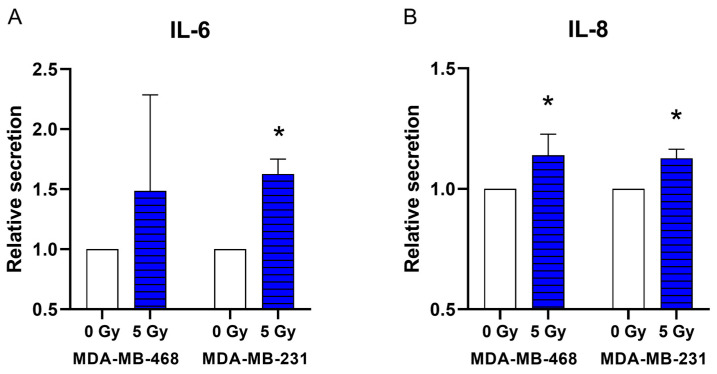
IL-6 (**A**) and IL-8 (**B**) secretion in the triple-negative cell lines MDA-MB-468 and MDA-MB-231 48 h after 5 Gy irradiation. The secretion of IL-6 and IL-8 in cell lines were measured with and without irradiation using an enzyme-linked immunosorbent assay (ELISA). The control (0 Gy) was set as 1. Values are presented as a mean ± SD. * *p* ≤ 0.05.

ELISA measurements revealed an enhanced IL-6 secretion in MDA-MB-231 cells 48 h after irradiation with 5 Gy (*p* = 0.04; Figure 4A). Cell culture supernatants of MDA-MB-468 and MDA-MB-231 showed a significant increase in IL-8 secretion after irradiation with 5 Gy compared to the non-irradiated control (*p* = 0.04; Figure 4B). Ionizing radiation did not stimulate or decrease the secretion of IL-6 and IL-8 in any of the other cell lines.

### 3.5. IL-6 and IL-8 Stimulates ATX Expression in ADSC

**Figure 5:**
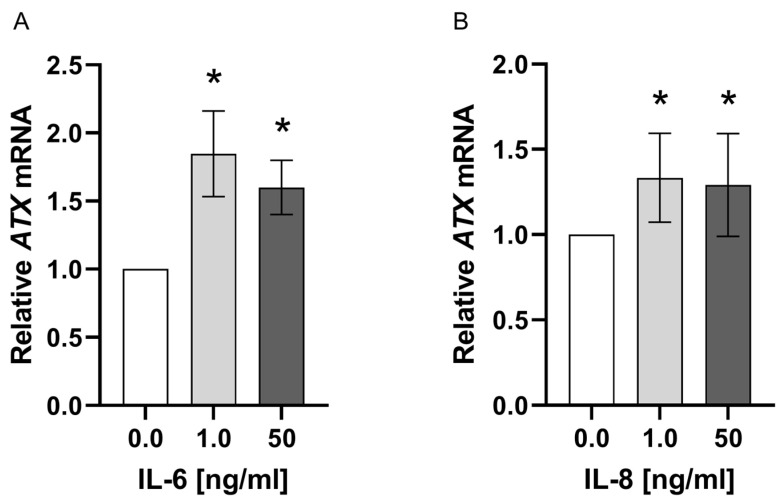
Alterations in *ATX* expression on the mRNA level of ASC/TERT1 cells 24 h after stimulation with IL-6 (**A**) and IL-8 (**B**). The columns show the mean relative *ATX* expression (y-axis) in varying concentrations of IL-6 and IL-8 (x-axis) compared to *YWHAZ* and to the control group. Values were calculated using the 2^−ΔΔCT^ method, where 2^−ΔΔCT^ of control = 1. * *p* < 0.05 (Mann–Whitney U test).

The ASC/TERT1 cells were stimulated with varying concentrations of IL-6 and IL-8 (1.0 ng/mL, 50 ng/mL). *ATX* expression was examined on the mRNA level 24 h after stimulation. *ATX* expression was significantly increased with 1.0 and 50 ng/mL stimulation compared to the non-stimulated control group (*p* = 0.04; Figure 5). No further increase could be achieved with 50 ng/mL compared to 1.0 ng/mL.

## 4. Discussion

Despite the continuous progress in the various treatment modalities for breast cancer, some patients develop therapy resistance and succumb to their disease. Radiation resistance is a critical aspect influencing treatment outcomes in breast cancer. Previous studies suggested the involvement of the ATX-LPA axis in protecting cancer cells from radiotherapy [2,22,25]. Understanding the mechanisms underlying ATX-induced radiotherapy resistance in breast cancer is important for the development of new therapeutic strategies. Targeting ATX or the downstream signaling pathways involved in LPA-mediated effects could potentially sensitize breast cancer cells to radiation and improve treatment outcomes. However, as breast cancer is a very inhomogeneous type of cancer with numerous subtypes, it is essential to clarify in which subtypes the ATX-LPA axis is relevant in the context of radiotherapy. As previous studies have focused on the role of adipose tissue or Specific tumor cell lines, this study concentrated on the influence of the ATX-LPA axis in several different breast cancer cell lines and their interaction with ADSC after irradiation. For the experiments, we selected the following breast cancer cell lines that can be assigned to different breast cancer subtypes, according to Dai et al. [27]: triple-negative A (MDA-MB-468), triple-negative B (MDA-MB-231), HER2-positive (SKBR-3), luminal A (MCF-7), and luminal B (BT-474). The non-tumorigenic epithelial mammary cell line MCF-10A served as the control.

In the present study, 5 Gy ionizing radiation reduced cell survival in all breast cancer cell lines and MCF-10A cells after 48 h. The direct effect of irradiation on cell survival was most pronounced in the triple-negative cell lines, which had the highest proliferation rate and showed the lowest in cell survival, indicating that the triple-negative cells are not, per se, more resistant to irradiation in in vitro laboratory conditions, as observed in vivo. In breast cancer treatment, the breast cancer subtype significantly impacts the response to radiation therapy, influencing outcomes such as local recurrence, survival, and response to treatment for metastatic disease [28,29,30]. Compared with other breast cancer subtypes, triple-negative breast cancer is more resistant to ionizing radiation, thus the triple-negative breast cancer cells acquire mechanisms for radiotherapy resistance [31]. However, radiation therapy is still a valuable component of the treatment plan for triple-negative breast cancer. In addition to improving treatment outcomes by a modulation of radiation-induced biological effects on breast cancer cells, current research is increasingly focusing on the tumor microenvironment and its role in promoting radioresistance [32,33,34]. Thereby, inflammation, extracellular matrix remodeling, immunological changes, and cancer-associated fibroblast modulation induced by tumor irradiation might play a key role in tumor spread and recurrence [32]. Adipose tissue in the tumor environment is also considered to play a decisive role in the development of radiation resistance. Meng et al. described that radiotherapy-induced damage to adipose tissue promotes ATX-LPA signaling, resulting in a feed-forward inflammatory cycle induced by the adipose tissue that potentially protects tumor cells from subsequent irradiation [26]. Further, ATX/LPA stimulates tumor-promoting cellular functions in breast cancer cells, particularly in triple-negative cell lines [17]. The data from our study suggested an influence of IL-6 and IL-8 on *ATX* expression in ADSC.

**Figure 6:**
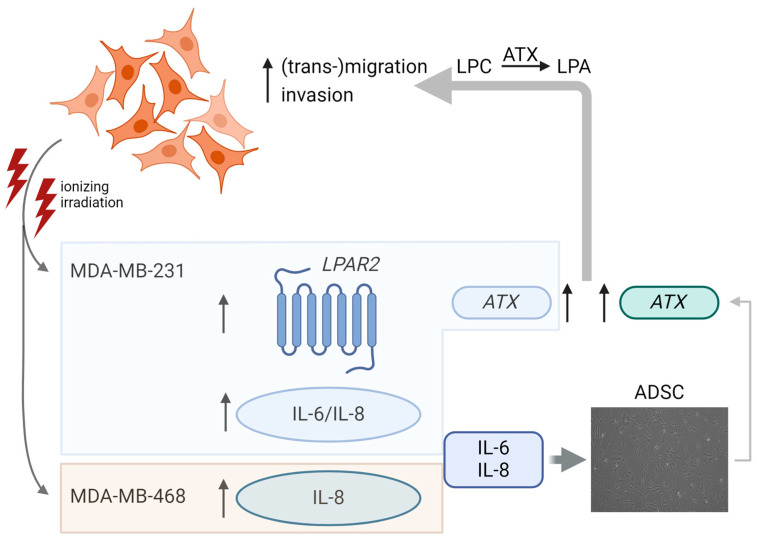
Ionizing radiation of triple-negative breast cancer cells induced enhanced expression of lysophosphatidic acid receptor (LPAR2) and autotaxin (ATX), and increased secretion of interleukin (IL)-6 and IL-8 in MDA-MB-231 cells. Additionally, enhanced secretion of IL-8 was observed in MDA-MB-468 cells. IL-6 and IL-8 could in turn stimulate ATX expression in ADSC in the tumor microenvironment. Created in BioRender. Kengelbach-Weigand, A. (2024) BioRender.com/j66o898.

First, we examined the influence of irradiation on *ATX* expression in breast cancer cells. In accordance with the literature, the breast cancer cells expressed little or virtually no *ATX*, and an *ATX* expression on the mRNA level could only be detected in the MDA-MB-231 cells. A previous study found no response to a single dose of 0.75 or 1 Gy irradiation in a triple-negative cell line (Hs578T) [35]. In MDA-MB-231 cells, 1 × 5 Gy irradiation promoted a threefold increase in *ATX* expression, suggesting considerable cell type-specific differences. Additionally, we investigated whether irradiation leads to an up- or down-regulation of *LPAR* in the various cell lines. Particularly *LPAR1–3* expression has been detected in breast cancer tissues [18]. Thereby, LPAR1–3 have been implicated in various aspects of breast cancer progression, including proliferation, migration, invasion, and metastasis [15,17,36]. LPAR6, on the other hand, is considered to have a protective effect and might act as a tumor suppressor in breast cancer [37]. Low expression of *LPAR6* in breast cancer tissue was correlated with poor prognosis [38]. In comparison between different breast cancer cell lines, the triple-negative cell lines MDA-MB-468 and MDA-MB-231 showed a lower relative *LPAR6* RNA expression, whereas *LPAR6* RNA expression was higher in luminal A breast cancer cell lines [37]. In the present study, we did not observe a significant increase or decrease in mRNA expression of *LPAR1*, *LPAR3*, and *LPAR6* RNA in breast cancer cells after irradiation. However, our data revealed an upregulation of *LPAR2* in MDA-MB-231 cells after 5 Gy irradiation. Radiation therapy induces DNA damage in cancer cells, which triggers cellular responses aimed at repairing the damage or inducing cell death. LPAR2 signaling has been implicated in promoting DNA repair mechanisms, thereby facilitating the repair of radiation-induced DNA damage and promoting increased resistance to radiation induced apoptosis [2,39,40,41]. Further, LPAR2 has been associated with a key role in signaling cytokine secretion [42]. A previous study proposed that LPAR2 is the most effective mediator linking LPA to the secretion of IL-6 and IL-8 [43]. Our results showed both an increased *LPAR2* expression and enhanced IL-6 and IL-8 secretion of MDA-MB-231 cells. Promoting the secretion of cytokines is a key component in radiotherapy. However, it can influence the tumor microenvironment and support tumor growth and metastasis. Previous findings showed that irradiation of tumor-associated fat pads increased cytokine secretion, including IL-6 [26]. We found enhanced secretion of IL-8 in both triple-negative breast cancer cell lines MDA-MB-231 and MDA-MB-468 after irradiation. In addition, particularly the IL-6 secretion of MDA-MB-231 cells was activated by the irradiation. Irradiation did not promote IL-6 or IL-8 secretion of the other cell lines. A previous study using high irradiation doses of 9 and 23 Gy also showed a stronger irradiation-related inflammatory response of MDA-MB-231 cells compared to MCF-7 cells [44]. Further, a comparison of the cytokine profile of conditioned medium from different tumor cell lines showed relatively poor cytokine secretion of MCF-7 cells [45]. The observed differences between triple-negative and the other cell lines may be due to their reliance on cytokines or growth factors instead of hormones for growth and survival. Inflammatory molecules such as IL-6 and IL-8 are secreted by the tumor cells themselves or by the surrounding stromal cells and adipose tissue, enhancing tumor growth and metastasis [46,47,48,49,50]. Thereby, IL-6 was shown to be involved in lymphangiogenesis in triple-negative breast cancer, leading to tumor growth and metastasis [51]. Another study reported the induction of a mesenchymal-like phenotype in triple-negative breast cancer cells by IL-8 and its essential role in epithelial-mesenchymal transition [52]. Recent findings described the involvement of IL-8 in proliferation and migration of tumor cells and the important role of IL-8 in crosstalk between triple-negative tumor cells and the tumor stroma, including fibroblasts and macrophages [47]. Further, IL-8 has been suggested as a major mediator to activate breast adipocytes, enhancing their paracrine protumorigenic effects [46]. The irradiation-induced increase in IL-6 and IL-8 secretion of the triple-negative cells in the present study might therefore have both a direct effect on the tumor cells and on the surrounding fat tissue. To investigate the role of the ATX-LPA axis in this context, we stimulated ADSC with different concentrations of IL-6 and IL-8. We found a stimulation of *ATX* expression in ADSC, which was higher after stimulation with IL-6. The different concentrations did not exhibit a dose-response to IL-6 and IL-8 in ASC/TER1 cells. This indicates that these cells might be highly sensitive to IL-6 and IL-8 so that even low concentrations seem to be sufficient to saturate the cellular response. Previous studies described an influence of *ATX* expression by certain inflammatory cytokines. Thereby, tumor necrosis factor alpha (TNF-α) induced *ATX* expression in mammary adipose tissue [22]. IL-6 was shown to stimulate the expression of *ATX* in fibroblasts, driving an amplification loop in human dermal fibroblasts in scleroderma fibrosis [53]. The present data also suggests an important role of the proinflammatory cytokines IL-6 and IL-8 in inducing *ATX* expression in ADSC. We therefore hypothesize that irradiation and ATX/LPA participate in an amplification loop in MDA-MB-231 cells, in which irradiation induces increased *ATX* and *LPAR2* expression. Further, irradiation increased interleukin secretion in triple-negative breast cancer cell lines MDA-MB-231 and MDA-MB-468. IL-6 and IL-8 in turn might induce *ATX* expression in ADSC in the tumor microenvironment (Figure 6). ATX further catalyzes LPA production, leading to increased migration and invasiveness in triple-negative breast cancer cells [17] and to an autocrine feedback loop based on proinflammatory signaling and ATX production within the adipose tissue [22].

A limitation of the study is the usage of a single exposure to irradiation, contrary to the clinical situation that relies on multiple fractions of radiation. However, previous findings suggested only a minor difference in cytokine secretion between a single dose compared with multiple fractions of irradiation [45]. Moreover, a previous study investigated the influence of the cytokine profile of breast cell lines after higher radiation doses (9 and 23 Gy). Thereby, in some cases, the lower dose resulted in higher cytokine secretion, whereas in other cases, the higher dose did. However, the trend was generally the same for both doses. Hence, the dose-effect seemed to less affect the cytokine secretion compared to the induction of cell death [44]. However, to further enhance the relevance and translatability of the present findings, future studies should explore the effects of higher varying irradiation doses on cellular responses. Further limitations of the present study are the lack of evidence on protein level for both *ATX* and *LPAR2* expression upon irradiation and the lack of translational evidence, such as preliminary data from patient tumor samples, to validate our in vitro findings. Additionally, it shows predominantly descriptive data, as it does not display cellular interactions between TNBC cell lines and ADCS, and it contains only limited functional analyses including mechanistic experiments. The absence of three-dimensional (3D) models that incorporate the tumor microenvironment and stromal compartments in the culture system to capture the complex tumor-stroma signaling is also a limitation of the study. This study was designed as an exploratory investigation and is the first to consider an interplay between breast cancer cells, irradiation and ATX focusing on IL-6 and IL-8 without the confounding effects of other cytokines present in the tumor microenvironment after irradiation in co-culture experiments. Further studies should include primary tumor samples and 3D models or patient-derived organoids and study the complete system, including additional cytokines, cell types, co-culture systems and functional analyses, aiming to provide more translational evidence and a more comprehensive understanding of the complex interactions between ATX/LPA, and radiotherapy resistance in breast cancer.

## 5. Conclusions

Our data showed an influence of ionizing irradiation on the ATX-LPA axis and on interleukin secretion only in triple-negative cell lines. IL-6 and IL-8 might in turn stimulate ATX expression in ADSC in the tumor microenvironment. Further exploration of the interplay between irradiation, the ATX-LPA axis, and inflammatory cytokines may guide the development of new personalized treatment paradigms for overcoming radioresistance in breast cancer therapy.

## Figures and Tables

**Table 1:** Primer sequences.

Gene	Forward	Reverse
*ENPP2*	TGAATCATCTCCTGCGCACT	ATCCAACTTGTTCTTTGGCTCT
*LPAR1*	TTTATGAAGCTCCCCATCCACC	TGAACACGCCCCAGAACTAC
*LPAR2*	TACCGAGAGACCACGCTCAG	GCCTAAACCATCCAGGAGCA
*LPAR3*	GAGTTTCCTGGGGGAATTTTGC	ACGTTCTCTCACTGTTCAGCA
*LPAR6*	TGGGTTGGACTCGTTGACTG	TTCCGCTGGGTTCTTCAACA
*hu YWHAZ*	ATGAGCTGGTTCAGAAGGCC	AAGATGACCTACGGGCTCCT

## Data Availability

The original contributions presented in the study are included in the article, further inquiries can be directed to the corresponding author.
